# Is the perceived neighborhood built environment associated with domain-specific physical activity in Latin American adults? An eight-country observational study

**DOI:** 10.1186/s12966-020-01030-6

**Published:** 2020-10-01

**Authors:** Gerson Ferrari, André Oliveira Werneck, Danilo Rodrigues da Silva, Irina Kovalskys, Georgina Gómez, Attilio Rigotti, Lilia Yadira Cortés Sanabria, Martha Yépez García, Rossina G. Pareja, Marianella Herrera-Cuenca, Ioná Zalcman Zimberg, Viviana Guajardo, Michael Pratt, Carlos Cristi-Montero, Fernando Rodríguez-Rodríguez, Adilson Marques, Ester Cerin, Delfien Van Dyck, Carlos Pires, Mauro Fisberg, on behalf of the ELANS Study Group

**Affiliations:** 1 Laboratorio de Ciencias de la Actividad Física, el Deporte y la Salud, Facultad de Ciencias Médicas, Universidad de Santiago de Chile, USACH, Las Sophoras 175, Estación Central, Santiago, Chile; 2 Department of Nutrition, School of Public Health, Universidade de São Paulo (USP), São Paulo, Brazil; 3 Department of Physical Education, Federal University of Sergipe – UFS, São Cristóvão, Brazil; 4 Carrera de Nutrición, Facultad de Ciencias Médicas, Pontificia Universidad Católica Argentina, Buenos Aires, Argentina; 5 Departamento de Bioquímica, Escuela de Medicina, Universidad de Costa Rica, San José, Costa Rica; 6 Centro de Nutrición Molecular y Enfermedades Crónicas, Departamento de Nutrición, Diabetes y Metabolismo, Escuela de Medicina, Pontificia Universidad Católica, Santiago, Chile; 7 Departamento de Nutrición y Bioquímica, Pontificia Universidad Javeriana, Bogotá, Colombia; 8 Colégio de Ciencias de la Salud, Universidad San Francisco de Quito, Quito, Ecuador; 9 Instituto de Investigación Nutricional, La Molina, Lima, Peru; 10 Centro de Estudios del Desarrollo, Universidad Central de Venezuela (CENDES-UCV)/Fundación Bengoa, Caracas, Venezuela; 11 Departamento de Psicobiologia, Universidade Federal de São Paulo, São Paulo, Brazil; 12 Institute for Public Health, University of California San Diego, La Jolla, CA USA; 13 IRyS Group, Physical Education School, Pontificia Universidad Católica de Valparaíso, Valparaíso, Chile; 14 CIPER, Faculdade de Motricidade Humana Universidade de Lisboa, Lisbon, Portugal; 15 ISAMB, Universidade de Lisboa, Lisbon, Portugal; 16 Mary MacKillop Institute for Health Research, Australian Catholic University, Melbourne, Australia; 17 School of Public Health, The University of Hong Kong, Pokfulam, Hong Kong, SAR China; 18 Department of Movement and Sports Sciences, Faculty of Medicine and Health Sciences, Ghent University, Ghent, Belgium; 19 Center for Research in Neuropsychology and Cognitive and Behavioral Intervention (CINEICC), Faculty of Psychology and Educational Sciences, University of Coimbra, Coimbra, Portugal; 20 Instituto Pensi, Fundação José Luiz Egydio Setubal, Hospital Infantil Sabará, São Paulo, Brazil; 21 Departamento de Pediatria da Universidade Federal de São Paulo, São Paulo, Brazil

**Keywords:** Epidemiology, Active transportation, Physical activity, Neighborhood built environment, Latin America

## Abstract

**Background:**

Characteristics of the neighborhood built environment are associated with physical activity (PA). However, few studies with representative samples have examined environmental correlates of domain-specific PA in Latin America. We examined the associations of the perceived neighborhood built environment with domain-specific PA in a large sample of adults from eight Latin American countries.

**Methods:**

This study examined data from 8185 adults (aged 18–65 years) from eight Latin American countries. The Neighbourhood Environment Walkability Survey - Abbreviated (NEWS-A) scale was used to assess perceptions of land use mix–diversity, land use mix-access, street connectivity, walking/cycling facilities, aesthetics, safety from traffic, and safety from crime. Perceived proximity from home to public open spaces (metropolitan parks, playgrounds, public squares) and to shopping centers was also measured. Transport-related and leisure-time PA were assessed using the long form of the International Physical Activity Questionnaire. Both logistic and linear regression models were estimated on pooled data.

**Results:**

Perceptions of higher land use mix-access (OR: 1.40; 95% CI: 1.22,1.61), the existence of many alternative routes in the neighbourhood (1.12; 1.04,1.20), slow speed of traffic (1.19; 1.03,1.35) and few drivers exceeding the speed limits (1.09; 1.03,1.15) were associated with greater odds of reporting at least 10 min/week of transport-related PA. Perceptions of higher levels of land use mix-diversity, better aesthetics and greater safety from crime, the presence of crosswalks and pedestrian signals, and greater proximity of shopping centers were associated with more min/week of transport-related PA. Perceptions of higher land use mix-diversity (1.12; 1.05,1.20), higher land use mix-access (1.27; 1.13,1.43), more walking/cycling facilities (1.18; 1.09,1.28), and better aesthetics (1.10; 1.02,1.18) were associated with greater odds of engaging in at least 10 min/week of leisure-time PA versus none. Perceptions of higher land use mix-diversity were associated with more min/week of leisure PA.

**Conclusions:**

Different perceived neighborhood built environment characteristics were associated with domain-specific PA among adults from Latin America countries. Interventions designed to modify perceptions of the neighbourhood built environment might influence initiation or maintenance of domain-specific PA.

**Trial registration:**

ClinicalTrials. Gov NCT02226627. Retrospectively registered on August 27, 2014.

## Introduction

The health benefits of physical activity (PA) are well established. They include a lower prevalence of cardiovascular disease (CVD), cognitive impairment, depression, blood pressure, obesity, breast and colon cancer, stroke, and mortality [1]. To achieve these physical and mental health benefits, adults should perform at least 150 min of moderate-to-vigorous PA or at least 75 min of vigorous PA intensity throughout the week [1]. In Latin America, only 39.1% of adults meet the PA guidelines [2].

In recent years, Latin America has undergone an accelerated urbanization process, with significant demographic, epidemiological, and socioeconomic changes. These changes resulted in improvements in general health and education indicators but also in a decrease in PA [3]. Eight out of ten individuals in Latin American countries live in cities, making it the most urbanized region in the world [4]. Latin American cities are characterized by high population density, disorganized and heavy traffic, air and noise pollution, rising crime rates, high-income inequality, high levels of poverty, and population aging, all of which might inhibit PA [5, 6].

Studies on the associations between neighborhood built environment factors and PA has increased in recent years. Neighborhood aspects such as destination accessibility, street connectivity, recreational facilities, and public transport have been linked to PA among adults [7]. These studies suggest that neighborhood environmental correlates of PA depend on the PA domain. For example, factors such as street connectivity, land use and distance to local destination have been found to be associated with both transport and leisure-time PA, but other factors, such as proximity to parks and safety from crime tend to show stronger associations with leisure-time PA [8–10]. Nonetheless, most previous research was conducted in the USA, Europe, or Australia [11–16]. Considering the distinct features of Latin American cities, it is not possible to directly translate findings from other countries (e.g., the USA or European countries) to this region. More specificly examining how characteristics of the built environment are associated with PA in eight Latin American countries may provide useful insights for guiding public policies and strategies for PA promotion in this region. There are relatively few studies about these factors among cities in Latin America [17–19]. Only one of these existing Latin American studies used a representative sample of the urban population [19]. Salvo et al. found that walkability (an index incorporating residential density, retail area-to-land ratio, connectivity) was inversely related to total weekly minutes of moderate-to-vigorous PA and the number of transit routes in a 500-m buffer around participants’ homes was inversely related to total weekly minutes of moderate-to-vigorous PA among adults in Cuernavaca, Mexico [19]. The purpose of the present study was to examine the associations of perceived neighborhood built environment with domain-specific PA in representative samples of adults from eight Latin America countries by country and overall adjusted for sociodemographic variables.

## Methods

### Latin American study of nutrition and health

The Latin American Study of Nutrition and Health (*Estudio Latinoamericano de Nutrición y Salud*; ELANS) is an eight-country (Argentina, Brazil, Chile, Colombia, Costa Rica, Ecuador, Peru, and Venezuela), observational epidemiological study using a common design and comparable methods across countries. The study uses a large representative sample (15–65 years old) from these eight countries and focuses on urban populations [20]. Data collection dates ranged from September 2014 to February 2015. The overarching ELANS protocol was approved by the Western Institutional Review Board (#20140605) and although the study is not a clinical trial, the protocol is registered at ClinicalTrials.gov (#NCT02226627) in order to increase the transparency of the methods and results and avoid criticism about publication bias. Ethical approval was obtained from each local institutional review boards and participants` informed consent/assent was obtained.

The entire ELANS study consisted of 9218 (4409 men) participants who were chosen using a random complex, multistage sampling frame with a random selection of Primary Sampling Units (PSUs) and Secondary Sampling Units (SSUs). The participants were recruited from PSUs areas (e.g., counties, municipalities, neighborhoods, residential areas) within each selected city in each country. An “n” size proportional to population weight was used for the selection of PSUs. In this case, a simple random sampling of “n” with replacement was performed to adhere to the principle of statistical independence of the selection of the areas included in the PSU sample. For these random selections, the probability proportional to size method was applied. Thus, within each of the areas included in the PSU distribution, a representative sample of SSUs was randomly designated using the probability proportional to size method.

For the selection of households, we implemented a four-step, systematic randomization procedure by establishing a selection interval (k): a) the total urban population was used to proportionally describe the main regions and to select cities representing each region; b) the sampling points (survey tracts) of each city were randomly designated, and c) clusters of households were selected from each sampling unit; d) the designated respondent within each household was selected using the birthday method. Details about participant sampling and recruitment strategies have been published elsewhere [20, 21].

In each country, stratified recruitment of individuals was done across sex, age group (15–19.9, 20–34.9, 35–49.9, and 50–65 years), and socioeconomic level (low, medium, high). Socioeconomic levels were weighted according to the national indices of each country. The number of individuals required per socioeconomic level has been addressed in more detail elsewhere [20]. In total, 92 cities participated in this study (7 to 23 cities in each country) [22]. The required sample size was calculated using a power analysis with a 95% confidence level, a maximum error of 3.5%, and a survey design effect of 1.75, resulting in a required sample size of 9090.

For household selection within cities, the systematic randomization method was used. The exclusion criteria adopted were: a) pregnant and lactating women; b) persons with physical or mental disabilities; c) unsigned consent form; d) individuals living in non-family residential environments; and e) individuals who could not read. More information on the ELANS study is provided in Fisberg et al. [20]

The perceived neighborhood built environment and PA protocol used in ELANS includes self-reported data collected by questionnaires. In this study, the questionnaires (perceived neighborhood built environment and PA) were interviewer-administered during the home visit, and 8185 (18–65 years old) participants had complete data. We excluded adolescents (15 to 17 years old) from the analyses because the ELANS study did not include adolescents of all ages. Also, adolescents may have restricted independent mobility [23] that may yield different environment-PA associations than those observed in adults. In addition, PA guidelines for adolescents differ from those for adults [24].

### Perceived neighborhood built environment

To assess perceptions of the neighborhood built environment characteristics, the Neighborhood Environment Walkability Scale - Abbreviated (NEWS-A) [25] previously translated into Spanish and adapted for use in Latin America [14, 26]. Apart from translating the NEWS-A from English into the local language of the participating countries, scale adaptation also encompassed the addition of two items to the safety from crime subscale, an item measuring the proximity of shopping centers and three items gauging the proximity to three types of public open space (metropolitan parks, playgrounds and public squares) as shown in Table 1. The reliability and validity of NEWS-A have been evaluated in several countries with all included scales having test-retest reliability intraclass correlations > 0.50 [27, 28].

**Table 1:** Summary of environmental scales, items and Cronbach’s alpha

Scale	Items	Response Category	Cronbach’s alpha
Land use mix-diversity (mean of 23 items – the higher the score, the higher the diversity)	About how long would it take to get from your home to the nearest businesses or facilities listed below if you walked to them? Items: convenience/small grocery store, supermarket, blacksmith, fruit/vegetable market, laundry/dry cleaners, clothing store, post office, library, university/school, other educational centers, book store, fast food restaurant or street food, bakery/coffee shop, bank, non-fast food restaurant, video store, pharmacy/drug store, salon/barber shop, your job or school, public transport stop, park or square, gym or fitness facility	5-point scale: 5 min (5), 6–10 min (4), 11–20 min (3), 20–30 min (2), 30+ min (1)	0.934
Land use mix-access (mean of 5 items)	Stores are within easy walking distance of my home. It is easy to walk to a transit stop (bus, train) from my home. There are many places to go within easy walking distance of my home. The streets in my neighborhood are hilly, making my neighborhood difficult to walk in (reversed). There are major barriers to walking in my local area that make it hard to get from place to place (for example, freeways, railway lines, rivers) (reversed).	4-point scale: strongly disagree (1), disagree (2), agree (3), strongly agree (4)	0.697
Street connectivity (mean of 3 items)	The streets in my neighborhood do not have many cul-de-sacs (dead-end streets). The distance between intersections in my neighborhood is usually short (100 yards or less; the length of a football field or less). There are many alternative routes for getting from place to place in my neighborhood. (I don’t have to go the same way every time).	4-point scale: strongly disagree (1), disagree (2), agree (3), strongly agree (4)	0.432
Walking/cycling facilities (mean of 3 items)	There are sidewalks on most of the streets in my neighborhood. Sidewalks are separated from the road/traffic in my neighborhood by parked cars. There is a grass/dirt strip that separates the streets from the sidewalks in my neighborhood.	4-point scale: strongly disagree (1), disagree (2), agree (3), strongly agree (4)	0.624
Aesthetics (mean of 4 items)	There are trees along the streets in my neighborhood. There are many interesting things to look at while walking in my neighborhood. There are many attractive natural sights in my neighborhood (such as landscaping, views). There are attractive buildings/homes in my neighborhood.	4-point scale: strongly disagree (1), disagree (2), agree (3), strongly agree (4)	0.813
Safety from traffic (mean of 4 items)	There is so much traffic along nearby streets that it makes it difficult or unpleasant to walk in my neighborhood (reversed). The speed of traffic on most nearby streets is usually slow (50 km/h or less) Most drivers exceed the posted speed limits while driving in my neighborhood (reversed) There are crosswalks and pedestrian signals to help walkers cross busy streets in my neighborhood.	4-point scale: strongly disagree (1), disagree (2), agree (3), strongly agree (4)	0.192
Safety from crime (mean of 7 items)	My neighborhood streets are well lit at night. Walkers and bikers on the streets in my neighborhood can be easily seen by people in their homes. There is a high crime rate in my neighborhood (reversed). The crime rate in my neighborhood makes it unsafe to go on walks during the day (reversed). The crime rate in my neighborhood makes it unsafe to go on walks at night (reversed). The parks, public squares, green areas and recreation areas in my neighborhood are unsafe during the day (reversed).^a^ The parks, public squares, green areas and recreation areas in my neighborhood are unsafe at night (reversed).^a^	4-point scale: strongly disagree (1), disagree (2), agree (3), strongly agree (4)	0.806
Proximity of public open spaces^a^ (mean of 3 items)	How long, approximately, does it take you to walk from your home to the following types of public open spaces: metropolitan parks (large, with many green areas), playgrounds, public squares.	5-point scale: 5 min (1), 6–10 min (2), 11–20 min (3), 20–30 min (4), 30+ min (5)	0.695
Proximity of shopping centers^a^ (1 item)	How long, approximately, does it take you to walk from your home to shopping centers?	5-point scale: 5 min (1), 6–10 min (2), 11–20 min (3), 20–30 min (4), 30+ min (5)	–

^a^items not in the NEWS-A scale

The “street connectivity” and “safety from traffic” items were analyzed individually due to low internal consistency

The following characteristics were assessed: land use mix–diversity, land use mix-access, street connectivity, walking/cycling facilities, aesthetics, safety from traffic, and safety from crime. The land use mix-diversity scale is assessed by the perceived walking proximity from home to 23 different types of destinations, with responses ranging from 1 to 5-min walking distance (coded as 5, indicative of high walkability) to > 30-min walking distance (coded as 1, indicative of low walkability). The remaining six scales are average ratings of items answered on a four-point Likert scale (1 = strongly disagree to 4 = strongly agree). Scales were scored in a direction consistent with higher walkability and safety, with individual items reversed when necessary. The scales ‘street connectivity’ and ‘safety from traffic’ from NEWS-A were not included in the results due to low internal consistency. Instead, the individual items were analysed separately (Table 1).

### Physical activity assessment

Participants reported their PA levels by completing the long-form of the last 7 days, interview version of the International Physical Activity Questionnaire (IPAQ) in Spanish [29]. We adapted the IPAQ by using only the questions that covered the active transport-related and leisure-time domains [22]. The long-form IPAQ (last 7 days) has been validated internationally using CSA accelerometer (model 7164) to assess total PA in a variety of contexts (occupational, transport, household, leisure) and at different intensities (moderate, vigorous, walking, cycling) in individuals aged 18–65 years from 12 countries with Spearman’s correlation coefficients ranging from 0.46 to 0.96 [29].

The participants were instructed to report the frequency and duration (bouts of > 10 min) of PA in the domains of active transport and leisure. Specifically, the following questions were asked: a) “During the last 7 days, did you walk or use a bicycle (pedal cycle) for at least 10 minutes continuously to get to and from places?” (Yes, No); b) “During the last 7 days, on how many days did you walk or ride a bicycle for at least 10 minutes at a time to go from place to place?”; c) “How much time did you usually spend on one of those days to bicycle or walk from place to place?” These questions were asked separately for walking and cycling. Concerning leisure PA, the following questions were asked: a) “During the last 7 days, did you walk, or do any moderate or vigorous PA for at least 10 minutes continuously?” (Yes, No); b) “During the last 7 days, on how many days did you walk, or do moderate or vigorous PA for at least 10 minutes at a time in your leisure time?”; c) “How much time do you spend walking or doing moderate-to-vigorou PA in your leisure time?” Questions were asked separately for walking, moderate-intensity, and vigorous-intensity activities. Details on the assessment of PA by IPAQ have been published elsewhere [22].

IPAQ PA data are reported as min/week of walking, moderate and vigorous PA during leisure-time, and min/week of walking and cycling for transport-related purposes. Time (min/week) spent in each of the PA domains (i.e., transport-related and leisure-time) was calculated and used in analysis. In this study, we used transport-related PA (walking + bicycle) and leisure-time PA (walking + moderate + vigorous) separately.

An international comparison showed that IPAQ had comparable reliability and validity to other self-report PA assessment methods [29]. There is evidence for an acceptable degree of reliability and validity of the transport-related and leisure-time PA items of the IPAQ. For these items, intraclass correlation coefficients ranging from 0.42 to 0.75 have been reported [30, 31]. Also, moderate correlations (0.50–0.63) were found between diary measures of transport-related and leisure-time PA and the corresponding IPAQ items. In our study, the intraclass correlation coefficients of self-reported total weekly minutes of walking, transport-related, and leisure-time PA with accelerometry-estimated weekly minutes of moderate-intensity PA were between 0.24 and 0.35 (unpublished data).

### Statistical analysis

Statistical analyses were carried out with the software SPSS v.26. Means, standard deviations (SD), median (interquartile range: IQR), and percentages were computed, as appropriate, to describe the variables. Weighting was done according to sociodemographic characteristics, sex, socioeconomic level, and country [20].

Cronbach’s alpha was used to assess the internal consistency of the neighborhood environment characteristics’ scales. Because the PA variables were positively skewed and had a large number of zeros, two different multilevel regression models, with the country as the second level, were used to estimate the associations of neighborhood characteristics with PA (transport-related and leisure-time): a logistic regression model (odds ratio: OR; confidence interval 95%: 95%CI) with a binary dependent variable (0 = “<10 minutes of PA/week”, 1 = “≥10 minutes of PA/week”) followed by a linear model with the min/week of PA as the dependent variable. The linear regression model (*β*; 95%CI) was estimated using data from the respondents who reported ≥10 min of PA per week. Due to the non-normality of data, the minutes of PA were log-transformed for the linear models and the unstandardized coefficient values were back-transformed into min/week aiming to increase the clinical utility of the findings. Both models were adjusted for sex, age, socioeconomic level, and country. We present the overall (i.e., pooled) and country-specific results (Additional file 1: Table S1-S4). A significance level of 5% was adopted.

## Results

### Descriptive data

The total number of participants included in the ELANS study was 9218 (52.2% women) (aged 15.0–65.0 years). Overall, the final response rate was 88.8%. The final sample with all complete data consisted of 8185 participants. The characteristics of the participantes are presented in Table 2. Overall, 53% of the sample consisted of females and the mean age was 37.4 (SD: 13.3) years. Mean transport-related and leisure-time PA were 153.9 (SD: 216.7) and 162.2 (SD: 272.7) min/week, respectively. Average transport-related PA ranged between 105.0 min/week (SD: 179.5) (Venezuela) and 214.3 min/week (SD: 259.1) (Costa Rica), while leisure-time PA ranged between 98.5 min/week (SD: 235.3) (Venezuela) and 315.5 min/week (SD: 321.6) (Ecuador).

**Table 2:** Demographic characteristics and physical activity (PA) of participants

Variables	Overall	Argentina	Brazil	Chile	Colombia	Costa Rica	Ecuador	Peru	Venezuela
Sample size (n)	8185	1137	1803	770	1105	710	668	979	1013
Sociodemographic									
Age, mean (SD)	37.4 (13.3)	38.4 (13.2)	37.9 (13.1)	38.0 (13.4)	38.3 (13.9)	37.0 (13.2)	35.9 (13.4)	35.9 (12.9)	36.5 (13.2)
Sex (%)									
Men	47.0	43.6	46.3	47.3	48.2	48.9	49.0	46.3	48.4
Women	53.0	56.4	53.7	52.7	51.8	51.1	51.0	53.7	51.6
Socioeconomic level (%)									
Low	52.0	47.4	46.2	46.0	63.0	33.0	51.2	47.6	77.9
Medium	38.4	47.2	45.2	44.7	31.6	53.2	35.9	31.7	17.2
High	9.6	5.4	8.6	9.4	5.4	13.8	12.9	20.7	4.9
Transportation PA (min/week)									
Mean (SD)	153.9 (216.7)	165.3 (237.5)	141.5 (205.1)	157.3 (221.8)	172.9 (235.2)	214.3 (259.1)	126.5 (157.9)	165.4 (207.9)	105.0 (179.5)
Median (IQR)	75.0 (15.0–180)	70.0 (0–210.0)	70.0 (0–175.0)	75.0 (20.0–180.0)	90.0 (30.0–210.0)	120.0 (40.0–280.0)	90.0 (40.0–140.0)	100.0 (40.0–210.0)	45.0(0–120.0)
Leisure PA (min/week)									
Mean (SD)	162.2 (272.7)	146.0 (253.1)	125.4 (252.6)	191.9 (291.7)	169.4 (272.9)	162.9 (269.8)	315.5 (321.6)	178.3 (273.2)	98.5 (235.3)
Median (IQR)	10.0 (0–20.0)	5.0 (0–10.0)	5.0 (0–10.0)	27.5 (10.0–120.0)	20.0 (0–220.0)	10.0 (0–10.0)	20.0 (5.0–200.0)	40.0 (0–210.0)	10.0 (0–10.0)

*PA* physical activity, *SD* standard deviation, *IQR* interquartile range

Table 3 shows the overall and country-specific descriptive statistics for the perceived environmental attributes. Land use mix-diversity (5-points scale from 1 to 5) was the highest in Colombia (mean: 3.1; SD: 0.7), Argentina (mean: 3.0; SD: 0.8) and Ecuador (mean: 3.0; SD: 0.6), and the lowest in Venezuela (mean: 2.4; SD: 0.8). The overall mean score was 2.8 (SD: 0.8).

**Table 3:** Overall and country-specific perceived-environment scores

	Overall	Argentina	Brazil	Chile	Colombia	Costa Rica	Ecuador	Peru	Venezuela
Sample size	8185	1137	1803	770	1105	710	668	979	1013
Land use mix-diversity (score 1–5)	2.8 (0.8)	3.0 (0.8)	2.6 (0.8)	2.6 (0.6)	3.1 (0.7)	2.8 (0.8)	3.0 (0.6)	2.7 (0.7)	2.4 (0.8)
Land use mix-access (score 1–4)	3.0 (0.4)	3.2 (0.4)	3.0 (0.4)	3.2 (0.4)	2.9 (0.4)	3.2 (0.4)	2.9 (0.4)	3.0 (0.4)	3.0 (0.4)
Walking/cycling facilities (score 1–4)	2.8 (0.6)	2.9 (0.5)	2.7 (0.6)	3.2 (0.6)	2.7 (0.5)	2.8 (0.8)	2.6 (0.4)	2.6 (0.7)	2.8 (0.6)
Aesthetics (score 1–4)	2.6 (0.7)	2.6 (0.7)	2.5 (0.7)	2.9 (0.8)	2.6 (0.6)	2.6 (0.7)	2.4 (0.6)	2.3 (0.7)	2.6 (0.7)
Safety from crime (score 1–4)	2.5 (0.6)	2.4 (0.5)	2.4 (0.6)	2.8 (0.6)	2.6 (0.5)	2.5 (0.6)	2.6 (0.5)	2.6 (0.5)	2.2 (0.6)
Proximity of public open spaces (score 1–5)	2.7 (1.1)	3.0 (1.0)	2.4 (1.0)	3.4 (0.8)	2.7 (1.0)	3.3 (0.9)	2.6 (0.9)	2.4 (1.0)	2.2 (1.1)
Proximity of shopping centers^**(1)**^	2.0 (1.3)	2.9 (1.5)	1.5 (0.9)	2.0 (1.1)	2.0 (1.1)	2.5 (1.4)	1.7 (1.0)	1.9 (1.2)	2.1 (1.4)
**Street connectivity items**^**(2)**^									
The streets in my neighborhood do not have many cul-de-sacs (dead-end streets).	2.5 (0.9)	2.6 (1.0)	2.7 (0.9)	2.6 (1.1)	2.5 (0.8)	2.4 (0.9)	2.3 (0.7)	2.3 (0.8)	2.5 (0.9)
The distance between intersections in my neighborhood is usually short (100 yards or less; the length of a football field or less).	2.8 (0.8)	3.0 (0.8)	2.7 (0.8)	3.0 (0.9)	2.9 (0.7)	2.9 (0.8)	2.8 (0.7)	2.9 (0.8)	2.7 (0.8)
There are many alternative routes for getting from place to place in my neighborhood. (I don’t have to go the same way every time.)	3.0 (0.8)	3.1 (0.8)	3.0 (0.8)	3.2 (0.8)	3.0 (0.7)	3.1 (0.8)	3.0 (0.7)	3.0 (0.7)	3.0 (0.8)
**Safety from traffic items**^**(2)**^									
There is so much traffic along nearby streets that it makes it difficult or unpleasant to walk in my neighborhood. (reversed)	2.4 (0.9)	2.3 (0.9)	2.3 (0.9)	2.4 (0.9)	2.5 (0.8)	2.3 (1.0)	2.5 (0.8)	2.6 (0.8)	2.4 (0.8)
The speed of traffic on most nearby streets is usually slow (50 km/h or less).	2.5 (0.8)	2.4 (0.8)	2.7 (0.8)	2.6 (0.9)	2.6 (0.7)	2.5 (0.9)	2.6 (0.7)	2.6 (0.7)	2.5 (0.8)
Most drivers exceed the posted speed limits while driving in my neighborhood. (reversed)	2.3 (0.8)	2.2 (0.8)	2.1 (0.8)	2.3 (0.9)	2.4 (0.8)	2.2 (0.9)	2.3 (0.8)	2.4 (0.8)	2.4 (0.8)
There are crosswalks and pedestrian signals to help walkers cross busy streets in my neighborhood.	2.4 (0.9)	2.4 (0.9)	2.6 (0.9)	2.8 (0.9)	2.3 (0.8)	2.3 (0.9)	2.4 (0.8)	2.2 (0.8)	2.1 (0.9)

Results presented as mean (standard deviation)

^(1)^5-point scale: 5 min (1), 6–10 min (2), 11–20 min (3), 20–30 min (4), 30+ min (5)

^(2)^4-point scale: strongly disagree (1), disagree (2), agree (3), strongly agree (4)

The overall mean scores (4-points scales from 1 to 4) of land use mix-access (mean: 3.0; SD: 0.4) and walking/cycling facilities (mean: 2.8; SD: 0.6), suggested relatively high perceived land use mix-access and lack of facilities for walking or cycling. The country-specific means were similar to the overall means for both scales (differences ≤0.2), except for the walking/cycling facilities score in Chile, where the mean score was the lowest (mean: 3.2: SD: 0.6). The overall scores of aesthetics (mean: 2.6; SD: 0.7) and safety from crime (mean: 2.5; SD: 0.6) were close to the center of the scales (4-points scales from 1 to 4). Perceived safety from crime was the lowest in Venezuela (mean: 2.2; SD: 0.6) and the highest in Chile (mean: 2.8; SD: 0.6). Chile was also the country with the highest aesthetics mean score (mean: 2.9; SD: 0.8) (Table 3).

The overall mean scores of proximity of public open spaces (mean: 2.7; SD: 1.1) and of shopping centers (mean: 2.0; SD: 1.3) (5-points scales from 1 to 5) indicated greater perceived proximity of public open spaces than to shopping centers. The differences between the two scores were particularly large in Chile (mean: 3.4; SD: 0.8 – proximity of public open spaces; mean: 2.0; SD: 1.1 – proximity of shopping centers) and small in Argentina (mean: 3.0; SD: 1.0 – proximity of public open spaces; mean: 2.9; SD: 1.5 – proximity of shopping centers). The mean scores of the items of street connectivity and safety from traffic were similar across all countries (differences ≤0.2) (Table 3).

### Associations of environmental perceptions with transport-related PA

Pooled estimated associations of perceived neighborhood built environment subscales with transport-related PA (logistic and linear regression) are shown in Table 4. Perceived land use mix-access (OR: 1.40; 95%CI: 1.22, 1.61) and the existence of many alternative routes in the neighbourhood (OR: 1.12; 95%CI: 1.04, 1.20) were associated with higher odds of reporting any transport-related PA (here defined as ≥10 min/week). Perceived slow speed of traffic (OR: 1.19; 95%CI: 1.03, 1.35) and few drivers exceeding the speed limits (OR: 1.09; 95%CI: 1.03, 1.15) were also related to higher odds of reporting any transport-related PA.

**Table 4:** Multilevel regression models for transport-related physical activity (PA)

	Logistic Regression^**(1)**^ Any transport-related PA (0 = <10 min/week, 1 ≥ 10 min/week)		Linear Regression^**(2)**^ Non-zero reported transport-related PA LOG10 (min/week) within participants with min/week ≥10	
	OR (95%CI)	*p*	*β* (95%CI)	*p*
**Independent variables**				
Land use mix-diversity (score 1–5) ^**(3)**^	0.93 (0.86,1.01)	0.067	**0.119 (0.036,0.192)**	**0.025**
Land use mix-access (score 1–4) ^**(3)**^	**1.40 (1.22,1.61)**	**< 0.001**	−0.003 (−0.032,0.026)	0.855
Walking/cycling facilities (score 1–4) ^**(3)**^	1.06 (0.96,1.16)	0.270	−0.001 (− 0.021,0.019)	0.923
Aesthetics (score 1–4) ^**(3)**^	1.02 (0.93,1.11)	0.714	**0.028 (0.010,0.046)**	**0.003**
Safety from crime (score 1–4) ^**(3)**^	1.07 (0.97,1.19)	0.176	**0.123 (0.044,0.181)**	**0.037**
Proximity of public open spaces (score 1–5) ^**(4)**^	1.00 (0.94,1.06)	0.898	0.003 (−0.009,0.016)	0.594
Proximity of shopping centers ^**(4)**^	1.01 (0.96,1.06)	0.621	**0.011 (0.001,0.021)**	**0.037**
**Street connectivity items** ^**(5)**^				
The streets in my neighbourhood do not have many cul-de-sacs (dead-end streets).	0.97 (0.91,1.03)	0.330	−0.001 (−0.014,0.011)	0.844
The distance between intersections in my neighbourhood is usually short (100 yards or less; the length of a football field or less).	0.99 (0.93,1.06)	0.838	−0.008 (− 0.023,0.006)	0.252
There are many alternative routes for getting from place to place in my neighbourhood. (I don’t have to go the same way every time.)	**1.12 (1.04,1.20)**	**0.004**	0.009 (−0.007,0.025)	0.249
**Safety from traffic items** ^**(5)**^				
There is so much traffic along nearby streets that it makes it difficult or unpleasant to walk in my neighbourhood (reversed).	0.99 (0.92,1.06)	0.706	0.007 (−0.007,0.021)	0.318
The speed of traffic on most nearby streets is usually slow (50 km/h or less).	**1.19 (1.03,1.35)**	**0.002**	0.001 (−0.013,0.015)	0.927
Most drivers exceed the posted speed limits while driving in my neighbourhood (reversed).	**1.09 (1.03,1.15)**	**0.017**	0.002 (−0.012,0.017)	0.765
There are crosswalks and pedestrian signals to help walkers cross busy streets in my neighbourhood.	0.95 (0.89,1.02)	0.134	**0.020 (0.007,0.033)**	**0.003**

*OR* odds ratio, *β* regression coefficient, *CI* confidence interval

^(1)^Multilevel logistic regression model (country as 2nd level) with transport-related physical activity time (0 = <10 min/week, 1 ≥ 10 min/week) as dependent variable, adjusted for sex, age, and socioeconomic level

^(2)^Multilevel linear regression model (country as 2nd level) with transport-related physical activity time (LOG10 (min/week)) as dependent variable in participants with transport-related physical activity ≥10 min/week, adjusted for sex, age, socioeconomic level

^(3)^higher scores indicate perception of higher land use mix-diversity, higher land use mix-access, more walking/cycling facilities, better aesthetics, and more safety from crime

^(4)^higher scores indicate greater proximity

^(5)^4-point scale: strongly disagree (1), disagree (2), agree (3), strongly agree (4)

The linear regression model showed that an increase of one point on the scale of land use mix-diversity was associated with a proportional increase of 1.32 min/week of transport-related PA (i.e., 32% increase, equivalent to 49.2 min/week; *β*_*log10*_: 0.119, 95%CI: 0.036, 0.192). Likewise, aesthetics was associated with a proportional increase 1.07 min/week (i.e., 7% increase, equivalent to 10.8 min/week; *β*_*log10*_: 0.028; 95%CI: 0.010, 0.046), and safety from crime was associated with a proportional increase 1.33 min/week (i.e., 33% increase, equivalent to 50.8 min/week; *β*_*log10*_: 0.123; 95%CI: 0.044, 0.181), greater proximity to shopping centers was associated with a proportional increase 1.03 min/week (i.e., 3% increase, equivalent to 4.6 min/week; *β*_*log10*_: 0.011; 95%CI: 0.011, 0.021), and the presence of crosswalks and pedestrian signals was associated with a proportional increase 1.05 min/week (i.e., 5% increase, equivalent to 7.7 min/week; *β*_*log10*_: 0.020; 95%CI: 0.007, 0.033) more minutes of transport-related PA (Table 4).

The country-specific results of associations of environmental perceptions with transport-related PA are available in Tables S1-S2 (Additional file 1). Different associations by country were observed between perceived neighborhood built environment and transport-related PA. Argentina was the country with the strongest associations between perceived aspects of the neighborhood built environment (land use mix-diversity, land use mix-access, aesthetics, safety from crime, public open spaces, streets in neighbourhood do not have many cul-de-sacs, the existence of many alternative routes in the neighbourhood, much traffic, and most drivers exceed the posted speed limits) and transport-related PA (Additional file 1: Tables S1-S2).

### Associations of environmental perceptions with leisure-time PA

In the pooled analyses, the odds of reporting any leisure PA were higher in participants perceiving higher land use mix-diversity (OR: 1.12; 95%CI: 1.05, 1.20), higher land use mix-access (OR: 1.27; 95%CI: 1.13, 1.43), more walking/cycling facilities (OR: 1.18; 95%CI: 1.09, 1.28), and better aesthetics (OR: 1.10; 95%CI: 1.02, 1.18). The linear regression analyses showed that the increase of one point in the perceived land use mix-diversity was associated with a proportional 1.34 more min/week of leisure PA in respondents who did any leisure PA (i.e., 34% increase, equivalent to 55.1 min/week; *β*_*log10*_: 0.127; 95%CI: 0.051, 0.193) (Table 5).

**Table 5:** Multilevel regression models for leisure physical activity (PA)

	Logistic Regression^**(1)**^ Any leisure-time PA (0 = < 10 min/week, 1 ≥ 10 min/week)		Linear Regression^**(2)**^ Non-zero reported leisure-time PA LOG10 (min/week) within participants with min/week ≥10	
	OR (95%CI)	*p*	*β* (95%CI)	*p*
**Independent variables**				
Land use mix-diversity (score 1–5) ^**(3)**^	**1.12 (1.05,1.20)**	**0.001**	**0.127 (0.051,0.193)**	**0.028**
Land use mix-access (score 1–4) ^**(3)**^	**1.27 (1.13,1.43)**	**< 0.001**	0.007 (−0.035,0.050)	0.740
Walking/cycling facilities (score 1–4) ^**(3)**^	**1.18 (1.09,1.28)**	**< 0.001**	−0.028 (− 0.057,0.002)	0.063
Aesthetics (score 1–4) ^**(3)**^	**1.10 (1.02,1.18)**	**0.016**	−0.007 (− 0.033,0.020)	0.630
Safety from crime (score 1–4) ^**(3)**^	1.04 (0.95,1.13)	0.400	−0.011 (− 0.042,0.019)	0.462
Proximity of public open spaces (score 1–5) ^**(4)**^	0.98 (0.93,1.03)	0.324	0.005 (−0.012,0.023)	0.545
Proximity of shopping centers ^**(4)**^	1.02 (0.98,1.07)	0.255	0.010 (−0.004,0.024)	0.160
**Street connectivity items** ^**(5)**^				
The streets in my neighbourhood do not have many cul-de-sacs (dead-end streets).	0.98 (0.94,1.03)	0.526	−0.014 (− 0.032,0.004)	0.120
The distance between intersections in my neighbourhood is usually short (100 yards or less; the length of a football field or less).	0.98 (0.92,1.04)	0.508	−0.006 (− 0.027,0.015)	0.557
There are many alternative routes for getting from place to place in my neighbourhood. (I don’t have to go the same way every time.)	1.02 (0.96,1.09)	0.509	0.015 (−0.008,0.038)	0.190
**Safety from traffic items** ^**(5)**^				
There is so much traffic along nearby streets that it makes it difficult or unpleasant to walk in my neighbourhood (reversed).	1.00 (0.94,1.06)	0.960	0.009 (−0.011,0.029)	0.367
The speed of traffic on most nearby streets is usually slow (50 km/h or less).	1.01 (0.96,1.07)	0.663	−0.007 (− 0.027,0.013)	0.511
Most drivers exceed the posted speed limits while driving in my neighbourhood (reversed).	1.05 (0.98,1.11)	0.154	0.001 (−0.020,0.022)	0.933
There are crosswalks and pedestrian signals to help walkers cross busy streets in my neighbourhood.	0.97 (0.92,1.02)	0.253	0.018 (−0.001,0.037)	0.066

*OR* odds ratio, *β* regression coefficient, *CI* confidence interval

^(1)^Multilevel logistic regression model (country as 2nd level) with leisure-time physical activity (0 = <10 min/week, 1 ≥ 10 min/week) as dependent variable, adjusted for sex, age, and socioeconomic level

^(2)^Multilevel linear regression model (country as 2nd level) with leisure-time physical activity (LOG10 (min/week)) as dependent variable in participants with leisure physical activity ≥10 min/week, adjusted for sex, age, socioeconomic level;

^(3)^higher scores indicate perception of higher land use mix-diversity, higher land use mix-access, more walking/cycling facilities, better aesthetics, and more safety from crime

^(4)^higher scores indicate greater proximity

^(5)^4-point scale: strongly disagree (1), disagree (2), agree (3), strongly agree (4)

The country-specific associations of environmental perceptions with leisure-time PA are shown in Tables S3-S4 (Additional file 1). Distinct associations by country were observed between perceived neighborhood built environment characteristics and leisure-time PA. Brazil was the country with the strongest associations between perceived aspects of the neighborhood built environment (land use mix-diversity, mix-access, walking/cycling facilities, the distance between intersections in the neighbourhood, speed of traffic, the presence of crosswalk and pedestrian signals, and proximity of shopping centers) and leisure-time PA (Additional file 1: Tables S3-S4).

## Discussion

The present study examined the associations between a wide range of perceived neighborhood built environment characteristics and domain-specific PA among adults from eight Latin American countries. Overall, perceived good access to services, many alternative routes to and from destinations, and low traffic speed were associated with higher odds of reporting any transport-related PA. In addition, perceived aesthetics, access to diverse destinations, safety from crime, presence of crosswalks and pedestrian signals, and proximity of shopping centers were positively associated with more weekly minutes of transport-related PA. The odds of reporting any leisure-time PA were higher among those who perceived their neighborhoods to be more aesthetically pleasing, having better access to diverse destinations and amenities, and better walking/cycling facilities. Only higher levels of perceived access to diverse destinations were associated with more time spent in leisure-time PA.

In line with the findings of our pooled analyses, the International Physical Activity Network (IPEN) study of adults from 17 cities in 12 countries, including three cities from Latin America (Bogota [Colombia], Curitiba [Brazil], Cuernavaca [Mexico]), reported positive associations of transport-related walking with perceived land use mix – access, land use mix - diversity, street connectivity, and aesthetics [9]. The IPEN Adult study also observed positive associations of objectively-assessed street intersection density, parks, and land use mix with transport-related PA [14, 32]. Support for the positive effects of street connectivity and access to destination on adults’ transport-related PA can be also found in recent studies from Europe and New Zealand [33, 34]. Two countries in our study (Argentina and Costa Rica) showed a negative association between land use mix – diversity and any transport-related PA. This could be due to various reasons specific to the local environment such as area-level socio-economic status or crime rates, factors that were not included in the regression models of land use mix - diversity. Alternatively, the actual level of access to various destinations might have been so high that many respondents could reach destinations with minimal walking In fact, recent studies have observed negative effects of extreme levels of access to destinations on transport-related walking [35, 36]. These patterns are more likely to occur in locations with high population and destination densities as in many Latin American cities [6, 37]. Unexpected relationships between the built environment, parks and transport PA were also observed in Cuernavaca, Mexico and attributed to the local context [14].

The IPEN Adult study also found positive associations between leisure-time PA and aesthetics and access to diverse destinations but did not show an association with walking/cycling infrastructure as in our study [8]. Similarly, no significant associations between aesthetics and weekly minutes of recreational walking were observed in the IPEN Adult Latin American countries. This suggests that, in the context of Latin America, aesthetics may promote the adoption of leisure-time PA but may not have a substantial impact on the accumulated amount of leisure-time PA. Interestingly, as in the IPEN Adult study [9], aesthetics were found to be positively related to transportation-PA in the pooled analyses, although this attribute is hypothesized to impact only leisure-time PA [38]. It may be that aesthetically pleasing environments and green spaces can act as motivators for engaging in or spending more time in active transport. Alternatively, the distinction between leisure and transport PA may be unclear in dense, diverse communities where many trips fulfill both purposes.

While previous studies have found inconsistent associations between safety and transport-related PA we observed positive associations between transport PA and safety from crime and traffic [9, 33]. Perhaps, the degree of danger from both crime and traffic in Latin American cities accounts for these observations [5, 6]. These relationships may be especially challenging to tease out in Latin America where crime, traffic, and access to public transport vary greatly by neighborhood and where lower income residents must engage in active transport regardless of safety issues because they have no other choice [39, 40]. As in previous studies we found that multiple aspects of the built environment are associated with walking for transport or leisure [8, 17, 34, 38]. These findings are not surprising given that walking is the most common form of leisure-time PA among adults in all countries, and in most Latin American cities walking is also an essential part of urban mobility [41]. Our results support thoughtful integration of efforts to improve conditions for walking with major public transit development such as Bus Rapid Transit systems (BRT) [42, 43]. We hypothesized that PA for transport could be associated with BRT use possibly because BRT is the fastest mode of transport, it has fixed stations, and its implementation is related to built-environment transformations. A commercial speed of 28 km/h allows for major time savings, especially on long trips [39], resulting from the presence of dedicated lanes and large stations with access to buses through multiple doors, and overtaking lanes that are used by express and local transit services. BRT construction is also associated with improved walking infrastructure (pedestrian bridges and wider sidewalks) [40]. These factors together may attract users to walk longer distances to access BRT stations rather than use other available modes of transportation. In Latin America, BRT systems have been widely implemented as a cost-effective solution for urban mobility [44], but they also have the potential to stimulate the use of active modes of transport and reduce private car use, thus promoting PA [45].

It should be emphasized that the role of the public health policies in urban health and urban environments is particularly important in Latin America due to the significant cross-cutting impacts on human health and well-being. Public health researchers in Latin America have a responsibility to engage with policy decision making processes, taking part in debate and providing evidence-based strategies to promote policies that positively impact social determinants of health and transport and leisure time PA. By doing so, they can contribute to increased levels of PA, decreased risks of chronic diseases, and enhanced health equity.

This study has several limitations. Only urban areas were included in the study, thus the samples are not nationally representative. The cross-sectional design precludes determination of causality even with careful adjustment for covariates [35, 46, 47]. The use of self-report measures for both neighborhood environments and PA must be considered in the interpretation of the findings. Cultural differences have been reported in interpretation of IPAQ, and the lack of precision of IPAQ compared with objective measures of PA could decrease the likelihood of significant associations with environmental attributes [48]. NEWS has been found to assess density and access to services more accurately in low-to-medium density urban environments [49]. This study does have a number of strengths. These include the examination of perceptions of urban environments across eight countries, a large number of environmental variables assessed using common environmental measures, the large sample size, and comparable data collection protocols, which are rare in studies from Latin America. By providing a unique Latin American dataset, the present study enables cross-country comparisons not previously possible.

## Conclusion

Perceived neighbourhood built environment characteristics are differentially associated with domain-specific PA in Latin America and can inform the development of PA promotion programs. Prospective studies of environmental characteristics and PA are needed, as well as evidence from intervention studies in order to further our understanding of these relationships and better inform public health policy and programs.

Improving perceptions of neighbourhood built environment as well as modifying the actual neighbourhood built environment could potentially increase PA among adults from Latin America.

## Supplementary information

**Additional file 1: Table S1.** Logistic regression models for transport-related physical activity (PA) by country. **Table S2.** Linear regression models for transport-related physical activity (PA) by country. **Table S3.** Logistic regression models for leisure-time physical activity (PA) by country. **Table S4.** Linear regression models for leisure-time physical activity (PA) by country

## Data Availability

The datasets generated and/or analyzed during the current study are not publicly available due the terms of consent/assent to which the participants agreed but are available from the corresponding author on reasonable request. Please contact the corresponding author to discuss availability of data and materials.
