# Supplementary material for: Is the perceived neighborhood built environment associated with domain-specific physical activity in Latin American adults? An eight-country observational study
Source: Int J Behav Nutr Phys Act. 2020 Oct 1;17:125. doi: 10.1186/s12966-020-01030-6 (PMC7528583; doi:10.1186/s12966-020-01030-6)
Supplement: Supplementary file 1 — Additional file 1: Table S1. Logistic regression models for transport-related physical activity (PA) by country. Table S2. Linear regression models for transport-related physical activity (PA) by country. Table S3. Logistic regression models for leisure-time physical activity (PA) by country. Table S4. Linear regression models for leisure-time physical activity (PA) by country [file 12966_2020_1030_MOESM1_ESM.doc]

Table S1. Logistic regression models for transport-related physical activity (PA) by country

| **Independent variables** | Argentina | |  | Brazil | |  | Chile | |  | Colombia | |  | Costa Rica | |  | Ecuador | |  | Peru | |  | Venezuela | |
| --- | --- | --- | --- | --- | --- | --- | --- | --- | --- | --- | --- | --- | --- | --- | --- | --- | --- | --- | --- | --- | --- | --- | --- |
|  | OR  (95%CI) | p |  | OR  (95%CI) | p |  | OR  (95%CI) | p |  | OR  (95%CI) | p |  | OR  (95%CI) | p |  | OR  (95%CI) | p |  | OR  (95%CI) | p |  | OR  (95%CI) | p |
| Land use mix-diversity  (score 1-5) **^(1)^** | 0.56  (0.46, 0.69) | <0.001 |  | 1.18  (1.01, 1.37) | 0.033 |  | 1.18  (0.84, 1.67) | 0.334 |  | 0.64  (0.50, 0.81) | <0.001 |  | 1.40  (1.02, 1.90) | 0.034 |  | 1.16  (0.73, 1.84) | 0.520 |  | 0.96  (0.70, 1.33) | 0.816 |  | 0.82  (0.68, 0.99) | 0.043 |
| Land use mix-access  (score 1-4) **^(1)^** | 1,46  (1.03, 2.07) | 0.033 |  | 1.10  (0.82, 1.47) | 0.524 |  | 1.12  (0.70, 1.79) | 0.631 |  | 3.19  (1.92, 5.31) | <0.001 |  | 1.26  (0.75, 2.10) | 0.387 |  | 0.60  (0.27, 1.35) | 0.214 |  | 1.24  (0.72, 2.14) | 0.444 |  | 1.64  (1.18, 2.28) | 0.003 |
| Walking/cycling facilities  (score 1-4) **^(1)^** | 0.98  (0.72, 1.32) | 0.871 |  | 1.20  (0.98, 1.48) | 0.079 |  | 1.04  (0.75, 1.44) | 0.819 |  | 0.81  (0.57, 1.14) | 0.222 |  | 0.96  (0.71, 1.29) | 0.788 |  | 1.29  (0.71, 2.34) | 0.411 |  | 1.44  (1.03, 2.01) | 0.033 |  | 1.05  (0.84, 1.31) | 0.690 |
| Aesthetics  (score 1-4) **^(1)^** | 1.40  (1.08, 1.81) | 0.010 |  | 0.85  (0.72, 1.00) | 0.053 |  | 1.07  (0.82, 1.40) | 0.617 |  | 1.15  (0.83, 1.59) | 0.394 |  | 1.16  (0.84, 1.60) | 0.365 |  | 1.00  (0.64, 1.56) | 0.996 |  | 2.01  (1.40, 2.88) | <0.001 |  | 0.87  (0.71, 1.07) | 0.191 |
| Safety from crime  (score 1-4) **^(1)^** | 1.60  (1.21, 2.12) | <0.001 |  | 1.35  (1.09, 1.67) | 0.006 |  | 0.70  (0.50, 0.97) | 0.031 |  | 0.78  (0.56, 1.10) | 0.156 |  | 0.98  (0.68, 1.41) | 0.899 |  | 0.77  (0.47, 1.27) | 0.309 |  | 0.90  (0.58, 1.38) | 0.627 |  | 1.08  (0.85, 1.38) | 0.535 |
| Proximity of public open spaces  (score 1-5) **^(2)^** | 1.16  (1.00, 1.34) | 0.048 |  | 0.93  (0.83, 1.05) | 0.246 |  | 0.79  (0.63, 0.99) | 0.043 |  | 1.02  (0.85, 1.22) | 0.816 |  | 1.11  (0.85, 1.46) | 0.432 |  | 1.11  (0.80, 1.53) | 0.542 |  | 0.89  (0.72, 1.10) | 0.293 |  | 1.03  (0.90, 1.18) | 0.698 |
| Proximity of shopping centers **^(2)^** | 1.00  (0.90, 1.11) | 0.984 |  | 1.01  (0.89, 1.15) | 0.887 |  | 0.96  (0.81, 1.13) | 0.591 |  | 1.08  (0.93, 1.54) | 0.326 |  | 0.86  (0.73, 1.03) | 0.097 |  | 1.40  (1.01, 1.93) | 0.042 |  | 1.01  (0.85, 1.20) | 0.910 |  | 0.98  (0.88, 1.09) | 0.752 |
| **Street connectivity items ^(3)^** | | |  |  |  |  |  |  |  |  |  |  |  |  |  |  |  |  |  |  |  |  |  |
| The streets in my neighbourhood do not have many cul-de-sacs (dead-end streets). | 0.82  (0.71, 0.95) | 0.008 |  | 0.99  (0.88, 1.13) | 0.935 |  | 0.97  (0.81, 1.16) | 0.734 |  | 1.03  (0.83, 1.27) | 0.810 |  | 0.88  (0.68, 1.14) | 0.348 |  | 0.95  (0.68, 1.31) | 0.738 |  | 1.15  (0.92, 1.45) | 0.230 |  | 1.11  (0.95, 1.29) | 0.175 |
| The distance between intersections in my neighbourhood is usually short (100 yards or less; the length of a football field or less). | 0.93  (0.78, 1.12) | 0.441 |  | 0.94  (0.82, 1.08) | 0.412 |  | 1.00  (0.80, 1.24) | 0.977 |  | 1.13  (0.88, 1.46) | 0.327 |  | 1.28  (0.96, 1.71) | 0.090 |  | 1.31  (0.91, 1.88) | 0.145 |  | 1.23  (0.96, 1.58) | 0.108 |  | 0.97  (0.81, 1.15) | 0.698 |
| There are many alternative routes for getting from place to place in my neighbourhood. (I don't have to go the same way every time.) | 1.34  (1.13, 1.60) | <0.001 |  | 0.99  (0.85, 1.15) | 0.872 |  | 1.16  (0.91, 1.48) | 0.243 |  | 1.20  (0.93, 1.54) | 0.168 |  | 1.01  (0.75, 1.35) | 0.946 |  | 1.23  (0.84, 1.80) | 0.296 |  | 0.91  (0.68, 1.22) | 0.529 |  | 1.17  (0.97, 1.41) | 0.110 |
| **Safety from traffic items ^(3)^** | | |  |  |  |  |  |  |  |  |  |  |  |  |  |  |  |  |  |  |  |  |  |
| There is so much traffic along nearby streets that it makes it difficult or unpleasant to walk in my neighbourhood (reversed). | 1.28  (1.07, 1.53) | 0.008 |  | 0.87  (0.75, 1.00) | 0.049 |  | 1.07  (0.87, 1.30) | 0.531 |  | 1.08  (0.86, 1.35) | 0.514 |  | 0.92  (0.71, 1.19) | 0.529 |  | 0.97  (0.69, 1.36) | 0.866 |  | 0.90  (0.69, 1.17) | 0.420 |  | 0.87  (0.73, 1.04) | 0.134 |
| The speed of traffic on most nearby streets is usually slow (50 km/h or less). | 0.93  (0.78, 1.10) | 0.404 |  | 0.92  (0.80, 1.05) | 0.204 |  | 0.93  (0.76, 1.14) | 0.480 |  | 0.90  (0.72, 1.12) | 0.346 |  | 0.71  (0.54, 0.93) | 0.012 |  | 1.06  (0.74, 1.50) | 0.764 |  | 0.87  (0.66, 1.14) | 0.305 |  | 0.92  (0.77, 1.10) | 0.357 |
| Most drivers exceed the posted speed limits while driving in my neighbourhood (reversed). | 0.74  (0.61, 0.91) | 0.004 |  | 1.29  (1.11, 1.49) | <0.001 |  | 1.01  (0.81, 1.27) | 0.913 |  | 1.10  (0.87, 1.39) | 0.445 |  | 1.22  (0.93, 1.59) | 0.155 |  | 1.21  (0.86, 1.70) | 0.283 |  | 0.97  (0.74, 1.28) | 0.841 |  | 1.13  (0.95, 1.36) | 0.175 |
| There are crosswalks and pedestrian signals to help walkers cross busy streets in my neighbourhood. | 0.97  (0.82, 1.14) | 0.690 |  | 0.97  (0.84, 1.12) | 0.691 |  | 1.04  (0.84, 1.27) | 0.731 |  | 0.97  (0.78, 1.19) | 0.737 |  | 0.93  (0.73, 1.18) | 0.548 |  | 0.75  (0.54, 1.05) | 0.089 |  | 1.06  (0.84, 1.35) | 0.606 |  | 0.97  (0.83, 1.14) | 0.727 |

OR: odds ratio; CI: confidence interval.

Multilevel logistic regression model with transport-related physical activity time (0=<10 min/week, 1≥10 min/week) as dependent variable, adjusted for sex, age, and socioeconomic level; **^(1)^** higher scores indicate perception of higher land use mix-diversity, higher land use mix-access, more walking/cycling facilities, better aesthetics, and more safety from crime; **^(2)^** higher scores indicate greater proximity; **^(3)^** 4-point scale: strongly disagree (1), disagree (2), agree (3), strongly agree (4).

Table S2. Linear regression models for transport-related physical activity (PA) by country

| **Independent variables** | Argentina | |  | Brazil | |  | Chile | |  | Colombia | |  | Costa Rica | |  | Ecuador | |  | Peru | |  | Venezuela | |
| --- | --- | --- | --- | --- | --- | --- | --- | --- | --- | --- | --- | --- | --- | --- | --- | --- | --- | --- | --- | --- | --- | --- | --- |
|  | β  (95%CI) | p |  | β  (95%CI) | p |  | β  (95%CI) | p |  | β  (95%CI) | p |  | β  (95%CI) | p |  | β  (95%CI) | p |  | β  (95%CI) | p |  | β  (95%CI) | p |
| Land use mix-diversity  (score 1-5) **^(1)^** | -0.077  (-0.107, 0.027) | 0.076 |  | 0.011  (-0.022, 0.044) | 0.524 |  | -0.021  (-0.088, 0.047) | 0.546 |  | -0.039  (-0.085, 0.007) | 0.097 |  | -0.023  (-0.076, 0.030) | 0.403 |  | 0.085  (0.014, 0.172) | 0.003 |  | -0.043  (-0.092, 0.007) | 0.093 |  | 0.042  (-0.008, 0.091) | 0.098 |
| Land use mix-access  (score 1-4) **^(1)^** | -0.009  (-0.094, 0.007) | 0.842 |  | 0.037  (-0.026, 0.100) | 0.254 |  | 0.042  (-0.056, 0.139) | 0.403 |  | -0.029  (-0.120, 0.063) | 0.536 |  | 0.017  (-0.075, 0.109) | 0.713 |  | -0.049  (-0.149, 0.052) | 0.341 |  | 0.030  (-0.056, 0.117) | 0.489 |  | 0.023  (-0.061, 0.108) | 0.588 |
| Walking/cycling facilities  (score 1-4) **^(1)^** | 0.054  (-0.016, 0.123) | 0.128 |  | 0.046  (0.020, 0.061) | 0.037 |  | 0.034  (-0.035, 0.102) | 0.333 |  | -0.064  (-0.127, 0.000) | 0.050 |  | -0.001  (-0.054, 0.052) | 0.970 |  | 0.016  (-0.059, 0.090) | 0.681 |  | 0.006  (-0.046, 0.057) | 0.834 |  | 0.037  (-0.019, 0.092) | 0.195 |
| Aesthetics  (score 1-4) **^(1)^** | 0.071  (0.011, 0.131) | 0.021 |  | 0.052  (0.015, 0.089) | 0.006 |  | 0.007  (-0.051, 0.064) | 0.815 |  | -0.004  (-0.064, 0.056) | 0.900 |  | 0.014  (-0.042, 0.070) | 0.622 |  | -0.005  (-0.063, 0.053) | 0.855 |  | 0.040  (-0.015, 0.095) | 0.154 |  | -0.007  (-0.061, 0.046) | 0.785 |
| Safety from crime  (score 1-4) **^(1)^** | -0.059  (-0.124, 0.006) | 0.074 |  | -0.015  (-0.062, 0.032) | 0.534 |  | -0.012  (-0.080, 0.056) | 0.725 |  | 0.091  (0.015, 0.212) | 0.004 |  | -0.057  (-0.122, 0.008) | 0.088 |  | -0.010  (-0.072, 0.053) | 0.764 |  | 0.031  (-0.038, 0.100) | 0.381 |  | 0.058  (-0.004, 0.120) | 0.069 |
| Proximity of public open spaces  (score 1-5) **^(2)^** | 0.008  (-0.027, 0.043) | 0.667 |  | -0.015  (-0.040, 0.010) | 0.253 |  | -0.042  (-0.088, 0.004) | 0.075 |  | 0.030  (-0.003, 0.063) | 0.078 |  | 0.043  (-0.002, 0.087) | 0.061 |  | 0.013  (-0.025, 0.052) | 0.501 |  | -0.008  (-0.041, 0.025) | 0.632 |  | 0.003  (-0.031, 0.037) | 0.858 |
| Proximity of shopping centers **^(2)^** | 0.068  (-0.019, 0.156) | 0.127 |  | 0.023  (-0.030, 0.076) | 0.397 |  | 0.133  (0.058, 0.208) | <0.001 |  | 0.000  (-0.067, 0.066) | 0.991 |  | -0.009  (-0.096, 0.077) | 0.833 |  | 0.054  (-0.010, 0.119) | 0.097 |  | 0.102  (0.038, 0.167) | 0.002 |  | 0.077  (0.007, 0.147) | 0.031 |
| **Street connectivity items ^(3)^** | | |  |  |  |  |  |  |  |  |  |  |  |  |  |  |  |  |  |  |  |  |  |
| The streets in my neighbourhood do not have many cul-de-sacs (dead-end streets). | -0.011  (-0.044, 0.022) | 0.507 |  | 0.028  (0.016, 0.034) | 0.044 |  | 0.019  (-0.017, 0.055) | 0.294 |  | -0.006  (-0.044, 0.033) | 0.774 |  | 0.024  (-0.025, 0.073) | 0.281 |  | 0.026  (-0.015, 0.068) | 0.214 |  | -0.006  (-0.041, 0.030) | 0.753 |  | 0.018  (-0.020, 0.056) | 0.361 |
| The distance between intersections in my neighbourhood is usually short (100 yards or less; the length of a football field or less). | -0.002  (-0.043, 0.039) | 0.925 |  | -0.004  (-0.034, 0.026) | 0.817 |  | -0.015  (-0.062, 0.031) | 0.513 |  | -0.026  (-0.072, 0.020) | 0.265 |  | 0.024  (-0.025, 0.073) | 0.341 |  | 0.021  (-0.026, 0.068) | 0.376 |  | 0.019  (-0.023, 0.061) | 0.367 |  | 0.081  (0.009, 0.151) | 0.005 |
| There are many alternative routes for getting from place to place in my neighbourhood. (I don't have to go the same way every time.) | -0.006  (-0.051, 0.039) | 0.795 |  | -0.019  (-0.013, 0.051) | 0.249 |  | -0.054  (-0.109, 0.000) | 0.052 |  | 0.012  (-0.037, 0.060) | 0.639 |  | 0.044  (-0.009, 0.098) | 0.104 |  | 0.035  (-0.014, 0.084) | 0.164 |  | -0.029  (-0.077, 0.018) | 0.228 |  | 0.026  (-0.023, 0.075) | 0.304 |
| **Safety from traffic items ^(3)^** | | |  |  |  |  |  |  |  |  |  |  |  |  |  |  |  |  |  |  |  |  |  |
| There is so much traffic along nearby streets that it makes it difficult or unpleasant to walk in my neighbourhood (reversed). | -0.024  (-0.067, 0.018) | 0.259 |  | 0.024 (-0.007, 0.054) | 0.131 |  | -0.007  (-0.048, 0.035) | 0.750 |  | 0.027  (-0.013, 0.068) | 0.184 |  | 0.032  (-0.011, 0.075) | 0.146 |  | -0.006  (-0.048, 0.037) | 0.796 |  | 0.011  (-0.030, 0.052) | 0.592 |  | -0.007  (-0.053, 0.039) | 0.763 |
| The speed of traffic on most nearby streets is usually slow (50 km/h or less). | -0.022  (-0.062, 0.019) | 0.296 |  | 0.001  (-0.029, 0.028) | 0.998 |  | 0.011  (-0.032, 0.053) | 0.620 |  | 0.028  (-0.013, 0.070) | 0.183 |  | 0.020  (-0.025, 0.065) | 0.388 |  | -0.030  (-0.076, 0.016) | 0.198 |  | 0.004  (-0.039, 0.047) | 0.865 |  | -0.013  (-0.056, 0.031) | 0.566 |
| Most drivers exceed the posted speed limits while driving in my neighbourhood (reversed). | 0.005  (-0.042, 0.052) | 0.829 |  | -0.012  (-0.043, 0.019) | 0.463 |  | 0.050  (0.005, 0.096) | 0.031 |  | -0.013  (-0.056, 0.029) | 0.530 |  | -0.005  (-0.050, 0.040) | 0.825 |  | 0.050  (0.008, 0.092) | 0.019 |  | -0.028  (-0.071, 0.015) | 0.206 |  | -0.047  (-0.089, 0.004) | 0.064 |
| There are crosswalks and pedestrian signals to help walkers cross busy streets in my neighbourhood. | 0.029  (-0.010, 0.067) | 0.151 |  | -0.009  (-0.039, 0.021) | 0.561 |  | 0.035  (-0.009, 0.078) | 0.115 |  | 0.041  (0.002, 0.079) | 0.039 |  | 0.012  (-0.029, 0.052) | 0.572 |  | 0.014  (-0.028, 0.055) | 0.520 |  | 0.008  (-0.030, 0.045) | 0.688 |  | 0.023  (-0.016, 0.062) | 0.253 |

CI: confidence interval.

Multilevel linear regression model with transport-related physical activity time (LOG10 (min/week)) as dependent variable in participants with transport-related physical activity ≥10 min/week, adjusted for sex, age, socioeconomic level; **^(1)^** higher scores indicate perception of higher land use mix-diversity, higher land use mix-access, more walking/cycling facilities, better aesthetics, and more safety from crime; **^(2)^** higher scores indicate greater proximity; **^(3)^** 4-point scale: strongly disagree (1), disagree (2), agree (3), strongly agree (4).

Table S3. Logistic regression models for leisure-time physical activity (PA) by country

| **Independent variables** | Argentina | |  | Brazil | |  | Chile | |  | Colombia | |  | Costa Rica | |  | Ecuador | |  | Peru | |  | Venezuela | |
| --- | --- | --- | --- | --- | --- | --- | --- | --- | --- | --- | --- | --- | --- | --- | --- | --- | --- | --- | --- | --- | --- | --- | --- |
|  | OR  (95%CI) | p |  | OR  (95%CI) | p |  | OR  (95%CI) | p |  | OR  (95%CI) | p |  | OR  (95%CI) | p |  | OR  (95%CI) | p |  | OR  (95%CI) | p |  | OR  (95%CI) | p |
| Land use mix-diversity  (score 1-5) **^(1)^** | 0.91  (0.76, 1.09) | 0.296 |  | 1.32  (1.15, 1.51) | <0.001 |  | 1.26  (0.95, 1.68) | 0.112 |  | 0.88  (0.73, 1.05) | 0.150 |  | 1.57  (1.27, 1.95) | <0.001 |  | 0.92  (0.66, 1.27) | 0.607 |  | 1.10  (0.87, 1.39) | 0.423 |  | 0.90  (0.73, 1.11) | 0.317 |
| Land use mix-access  (score 1-4) **^(1)^** | 1.08  (0.79, 1.47) | 0.619 |  | 1.64  (1.26, 2.13) | <0.001 |  | 1.41  (0.95, 2.10) | 0.086 |  | 1.25  (0.87, 1.80) | 0.229 |  | 1.35  (0.93, 1.95) | 0.114 |  | 0.81  (0.45, 1.47) | 0.488 |  | 1.27  (0.85, 1.88) | 0.242 |  | 0.80  (0.56, 1.15) | 0.234 |
| Walking/cycling facilities  (score 1-4) **^(1)^** | 0.91  (0.71, 1.18) | 0.495 |  | 1.41  (1.18, 1.70) | <0.001 |  | 1.18  (0.90, 1.55) | 0.239 |  | 1.13  (0.87, 1.46) | 0.335 |  | 1.03  (0.84, 1.27) | 0.787 |  | 0.81  (0.53, 1.25) | 0.337 |  | 1.21  (0.95, 1.54) | 0.121 |  | 1.10  (0.86, 1.42) | 0.440 |
| Aesthetics  (score 1-4) **^(1)^** | 1.16  (0.93, 1.44) | 0.193 |  | 1.07  (0.92, 1.25) | 0.357 |  | 0.99  (0.79, 1.24) | 0.931 |  | 1.27  (1.00, 1.62) | 0.048 |  | 1.04  (0.83, 1.30) | 0.737 |  | 0.97  (0.69, 1.35) | 0.838 |  | 1.59  (1.23, 2.07) | <0.001 |  | 1.10  (0.88, 1.39) | 0.394 |
| Safety from crime  (score 1-4) **^(1)^** | 1.01  (0.80, 1.28) | 0.906 |  | 1.09  (0.90, 1.32) | 0.359 |  | 0.84  (0.64, 1.11) | 0.222 |  | 0.82  (0.63, 1.05) | 0.116 |  | 1.06  (0.82, 1.36) | 0.675 |  | 1.08  (0.75, 1.56) | 0.670 |  | 0.98  (0.72, 1.34) | 0.895 |  | 1.22  (0.94, 1.60) | 0.142 |
| Proximity of public open spaces  (score 1-5) **^(2)^** | 1.00  (0.88, 1.13) | 0.967 |  | 0.96  (0.87, 1.07) | 0.473 |  | 0.92  (0.76, 1.11) | 0.375 |  | 1.09  (0.94, 1.25) | 0.246 |  | 0.84  (0.70, 1.01 | 0.051 |  | 1.05  (0.84, 1.33) | 0.648 |  | 0.95  (0.82, 1.11) | 0.532 |  | 1.10  (0.94, 1.28) | 0.232 |
| Proximity of shopping centers **^(2)^** | 0.97  (0.88, 1.07) | 0.530 |  | 1.11  (0.99, 1.24) | 0.070 |  | 1.07  (0.93, 1.23) | 0.333 |  | 1.08  (0.96, 1.22) | 0.220 |  | 1.28  (1.12, 1.44) | 0.034 |  | 0.97  (0.79, 1.18) | 0.738 |  | 1.01  (0.89, 1.14) | 0.915 |  | 1.07  (0.95, 1.20) | 0.257 |
| **Street connectivity items ^(3)^** | | |  |  |  |  |  |  |  |  |  |  |  |  |  |  |  |  |  |  |  |  |  |
| The streets in my neighbourhood do not have many cul-de-sacs (dead-end streets). | 0.94  (0.83, 1.06) | 0.305 |  | 1.07  (0.95, 1.20) | 0.263 |  | 0.98  (0.85, 1.13) | 0.788 |  | 1.14  (0.98, 1.33) | 0.095 |  | 0.85  (0.72, 1.01) | 0.064 |  | 1.01  (0.79, 1.29) | 0.922 |  | 1.03  (0.88, 1.22) | 0.707 |  | 0.94  (0.80, 1.11) | 0.484 |
| The distance between intersections in my neighbourhood is usually short (100 yards or less; the length of a football field or less). | 0.87  (0.75, 1.01) | 0.065 |  | 0.87  (0.77, 0.99) | 0.030 |  | 0.95  (0.78, 1.14) | 0.554 |  | 1.13  (0.94, 1.37) | 0.188 |  | 1.03  (0.85, 1.25) | 0.766 |  | 1.18  (0.90, 1.54) | 0.236 |  | 1.33  (1.10, 1.61) | 0.003 |  | 0.94  (0.78, 1.14) | 0.556 |
| There are many alternative routes for getting from place to place in my neighbourhood. (I don't have to go the same way every time.) | 0.97  (0.83, 1.13) | 0.697 |  | 1.02  (0.89, 1.16) | 0.813 |  | 1.12  (0.90, 1.39) | 0.296 |  | 1.10  (0.90, 1.33) | 0.364 |  | 0.94  (0.77, 1.16) | 0.564 |  | 1.10  (0.82, 1.47) | 0.519 |  | 1.12  (0.91, 1.39) | 0.289 |  | 0.99  (0.80, 1.22) | 0.921 |
| **Safety from traffic items ^(3)^** | | |  |  |  |  |  |  |  |  |  |  |  |  |  |  |  |  |  |  |  |  |  |
| There is so much traffic along nearby streets that it makes it difficult or unpleasant to walk in my neighbourhood (reversed). | 0.96  (0.82, 1.12) | 0.586 |  | 1.03  (0.91, 1.17) | 0.649 |  | 0.89  (0.76, 1.06) | 0.196 |  | 1.12  (0.95, 1.33) | 0.170 |  | 0.97  (0.82, 1.16) | 0.747 |  | 0.88  (0.68, 1.13) | 0.312 |  | 0.97  (0.80, 1.18) | 0.793 |  | 1.04  (0.85, 1.28) | 0.683 |
| The speed of traffic on most nearby streets is usually slow (50 km/h or less). | 0.98  (0.85, 1.14) | 0.792 |  | 1.16  (1.03, 1.31) | 0.015 |  | 0.89  (0.75, 1.05) | 0.173 |  | 1.09  (0.92, 1.29) | 0.311 |  | 0.91  (0.76, 1.08) | 0.273 |  | 0.87  (0.67, 1.14) | 0.316 |  | 1.09  (0.89, 1.33) | 0.404 |  | 0.94  (0.77, 1.14) | 0.536 |
| Most drivers exceed the posted speed limits while driving in my neighbourhood (reversed). | 0.98  (0.83, 1.17) | 0.849 |  | 0.93  (0.81, 1.06) | 0.250 |  | 1.10  (0.91, 1.32) | 0.326 |  | 0.99  (0.84, 1.18) | 0.945 |  | 0.98  (0.82, 1.18) | 0.869 |  | 1.03  (0.81, 1.32) | 0.797 |  | 0.82  (0.67, 1.00) | 0.054 |  | 1.04  (0.85, 1.26) | 0.734 |
| There are crosswalks and pedestrian signals to help walkers cross busy streets in my neighbourhood. | 1.08  (0.94, 1.24) | 0.293 |  | 0.82  (0.73, 0.93) | 0.002 |  | 1.02  (0.86, 1.22) | 0.785 |  | 0.88  (0.75, 1.03) | 0.108 |  | 1.12  (0.95, 1.31) | 0.175 |  | 0.90  (0.71, 1.16) | 0.426 |  | 0.98  (0.82, 1.17) | 0.841 |  | 1.03  (0.86, 1.23) | 0.751 |

OR: odds ratio; CI: confidence interval

Multilevel logistic regression model with leisure-time physical activity (0=<10 min/week, 1≥10 min/week) as dependent variable, adjusted for sex, age, and socioeconomic level; **^(1)^** higher scores indicate perception of higher land use mix-diversity, higher land use mix-access, more walking/cycling facilities, better aesthetics, and more safety from crime; **^(2)^** higher scores indicate greater proximity; **^(3)^** 4-point scale: strongly disagree (1), disagree (2), agree (3), strongly agree (4).

Table S4. Linear regression models for leisure-time physical activity (PA) by country

| **Independent variables** | Argentina | |  | Brazil | |  | Chile | |  | Colombia | |  | Costa Rica | |  | Ecuador | |  | Peru | |  | Venezuela | |
| --- | --- | --- | --- | --- | --- | --- | --- | --- | --- | --- | --- | --- | --- | --- | --- | --- | --- | --- | --- | --- | --- | --- | --- |
|  | β  (95%CI) | p |  | β  (95%CI) | p |  | β  (95%CI) | p |  | β  (95%CI) | p |  | β  (95%CI) | p |  | β  (95%CI) | p |  | β  (95%CI) | p |  | β  (95%CI) | p |
| Land use mix-diversity  (score 1-5) **^(1)^** | 0.096  (0.031, 0.152) | 0.003 |  | -0.030  (0.086, 0.027) | 0.301 |  | 0.035  (-0.056, 0.125) | 0.450 |  | 0.091  (0.025, 0.169) | 0.028 |  | 0.022  (-0.050, 0.095) | 0.543 |  | -0.033  (-0.103, 0.357) | 0.357 |  | 0.057  (-0.013, 0.127) | 0.113 |  | -0.028  (-0.119, 0.062) | 0.539 |
| Land use mix-access  (score 1-4) **^(1)^** | 0.087  (-0.019, 0.194) | 0.109 |  | -0.069  (-0.176, 0.037) | 0.203 |  | 0.019  (-0.115, 0.154) | 0.778 |  | -0.119  (-0.252, 0.014) | 0.080 |  | 0.088  (-0.044, 0.221) | 0.190 |  | 0.111  (-0.009, 0.231) | 0.070 |  | -0.001  (-0.124, 0.122) | 0.990 |  | -0.094  (-0.267, 0.079) | 0.286 |
| Walking/cycling facilities  (score 1-4) **^(1)^** | 0.016  (-0.078, 0.110) | 0.738 |  | -0.024  (-0.101, 0.054) | 0.549 |  | -0.025  (-0.120, 0.071) | 0.615 |  | 0.212  (0.071, 0.351) | 0.016 |  | 0.002  (-0.068, 0.072) | 0.952 |  | 0.067  (-0.025, 0.158) | 0.156 |  | -0.036  (-0.112, 0.039) | 0.347 |  | -0.049  (-0.153, 0.056) | 0.360 |
| Aesthetics  (score 1-4) **^(1)^** | -0.005  (-0.080, 0.070) | 0.859 |  | -0.005  (-0.071, 0.061) | 0.885 |  | 0.010  (-0.070, 0.089) | 0.812 |  | 0.059  (-0.031, 0.150) | 0.196 |  | -0.019  (-0.091, 0.054) | 0.614 |  | -0.046  (-0.118, 0.025) | 0.205 |  | -0.040  (-0.118, 0.039) | 0.311 |  | -0.008  (-0.117, 0.100) | 0.883 |
| Safety from crime  (score 1-4) **^(1)^** | 0.030  (-0.056, 0.116) | 0.494 |  | -0.004  (-0.081, 0.072) | 0.913 |  | -0.022  (-0.114, 0.070) | 0.636 |  | -0.047  (-0.135, 0.042) | 0.303 |  | -0.066  (-0.150, 0.019) | 0.126 |  | 0.048  (-0.026, 0.122) | 0.203 |  | 0.021  (-0.076, 0.118) | 0.676 |  | -0.038  (-0.160, 0.085) | 0.549 |
| Proximity of public open spaces  (score 1-5) **^(2)^** | 0.032  (-0.012, 0.075) | 0.155 |  | -0.003  (-0.045, 0.039) | 0.889 |  | -0.017  (-0.080, 0.047) | 0.605 |  | 0.012  (-0.036, 0.060) | 0.627 |  | 0.064  (0.004, 0.124) | 0.036 |  | 0.009  (-0.038, 0.055) | 0.717 |  | -0.013  (-0.060, 0.035) | 0.598 |  | 0.016  (-0.046, 0.078) | 0.611 |
| Proximity of shopping centers **^(2)^** | -0.014  (-0.121, 0.094) | 0.802 |  | 0.137  (0.048, 0.226) | 0.003 |  | 0.165  (0.061, 0.269) | 0.002 |  | 0.027  (-0.069, 0.123) | 0.528 |  | -0.108  (-0.221, 0.005) | 0.061 |  | 0.109  (0.051, 0.167) | 0.007 |  | -0.018  (0.111, 0.075) | 0.708 |  | -0.015  (-0.147, 0.118) | 0.828 |
| **Street connectivity items ^(3)^** | | |  |  |  |  |  |  |  |  |  |  |  |  |  |  |  |  |  |  |  |  |  |
| The streets in my neighbourhood do not have many cul-de-sacs (dead-end streets). | 0.006  (-0.036, 0.048) | 0.779 |  | 0.026  (-0.022, 0.073) | 0.295 |  | -0.020  (-0.068, 0.029) | 0.429 |  | -0.011  (-0.065, 0.043) | 0.680 |  | 0.010  (-0.046, 0.066) | 0.727 |  | 0.018  (-0.069, 0.032) | 0.482 |  | 0.167  (0.056, 0.272) | 0.008 |  | -0.020  (-0.094, 0.054) | 0.596 |
| The distance between intersections in my neighbourhood is usually short (100 yards or less; the length of a football field or less). | -0.023  (-0.074, 0.028) | 0.377 |  | -0.012  (-0.062, 0.073) | 0.622 |  | -0.018  (-0.081, 0.045) | 0.571 |  | -0.013  (-0.080, 0.054) | 0.706 |  | -0.046  (-0.108, 0.017) | 0.151 |  | 0.015  (-0.043, 0.073) | 0.612 |  | 0.064  (0.004, 0.125) | 0.038 |  | -0.029  (-0.117, 0.059) | 0.517 |
| There are many alternative routes for getting from place to place in my neighbourhood. (I don't have to go the same way every time.) | 0.050  (-0.004, 0.103) | 0.070 |  | -0.016  (-0.069, 0.038) | 0.563 |  | -0.066  (-0.142, 0.010) | 0.087 |  | 0.000  (-0.070, 0.070) | 0.996 |  | 0.055  (-0.013, 0.124) | 0.115 |  | 0.029  (-0.032, 0.089) | 0.352 |  | 0.033  (-0.035, 0.101) | 0.346 |  | 0.050  (-0.056, 0.156) | 0.355 |
| **Safety from traffic items ^(3)^** | | |  |  |  |  |  |  |  |  |  |  |  |  |  |  |  |  |  |  |  |  |  |
| There is so much traffic along nearby streets that it makes it difficult or unpleasant to walk in my neighbourhood (reversed). | 0.043  (-0.009, 0.095) | 0.107 |  | -0.018  (-0.070, 0.034) | 0.500 |  | -0.005  (-0.065, 0.054) | 0.860 |  | 0.033  (-0.027, 0.092) | 0.282 |  | -0.021  (-0.077, 0.034) | 0.446 |  | 0.015  (-0.038, 0.068) | 0.585 |  | 0.045  (-0.012, 0.103) | 0.124 |  | -0.024  (-0.110, 0.061) | 0.577 |
| The speed of traffic on most nearby streets is usually slow (50 km/h or less). | 0.000  (-0.051, 0.051) | 0.993 |  | -0.015  (-0.065, 0.035) | 0.545 |  | -0.027  (-0.087, 0.033) | 0.380 |  | -0.012  (-0.074, 0.049) | 0.695 |  | 0.027  (-0.029, 0.084) | 0.346 |  | -0.052  (-0.108, 0.004) | 0.068 |  | 0.013  (-0.046, 0.072) | 0.669 |  | -0.073  (-0.161, 0.016) | 0.106 |
| Most drivers exceed the posted speed limits while driving in my neighbourhood (reversed). | -0.026  (-0.084, 0.033) | 0.388 |  | -0.011  (-0.065, 0.042) | 0.674 |  | 0.096  (0.033, 0.159) | 0.003 |  | -0.043  (-0.103, 0.017) | 0.157 |  | 0.009  (-0.051, 0.068) | 0.779 |  | 0.034  (-0.018, 0.086) | 0.195 |  | -0.016  (-0.076, 0.044) | 0.607 |  | -0.001  (-0.093, 0.091) | 0.988 |
| There are crosswalks and pedestrian signals to help walkers cross busy streets in my neighbourhood. | 0.078  (0.030, 0.125) | <0.001 |  | -0.033  (-0.084, 0.017) | 0.194 |  | -0.010  (-0.072, 0.052) | 0.762 |  | 0.009  (-0.047, 0.065) | 0.756 |  | 0.034  (-0.018, 0.085) | 0.197 |  | 0.007  (-0.044, 0.058) | 0.790 |  | 0.037  (-0.015, 0.089) | 0.160 |  | 0.011  (-0.066, 0.089) | 0.771 |

CI: confidence interval.

Multilevel linear regression model with leisure-time physical activity (LOG10 (min/week)) as dependent variable in participants with leisure-time physical activity ≥10 min/week, adjusted for sex, age, socioeconomic level; **^(1)^** higher scores indicate perception of higher land use mix-diversity, higher land use mix-access, more walking/cycling facilities, better aesthetics, and more safety from crime; **^(2)^** higher scores indicate greater proximity; **^(3)^** 4-point scale: strongly disagree (1), disagree (2), agree (3), strongly agree (4).
